# Prevalence of *Encephalitozoon cuniculi* Infection in Guinea Pigs (*Cavia porcellus)* in Poland with Different Clinical Disorders—A Pilot Study

**DOI:** 10.3390/ani13121992

**Published:** 2023-06-14

**Authors:** Anna Wilczyńska, Renata Komsta, Mateusz Szadkowski, Jerzy Ziętek, Łukasz Adaszek

**Affiliations:** 1 Department of Epizootiology and Clinic of Infectious Diseases, Faculty of Veterinary Medicine of the University of Life Sciences in Lublin, ul. Głęboka 30, 20-612 Lublin, Poland; achantina@op.pl (J.Z.); lukasz.adaszek@up.lublin.pl (Ł.A.); 2 Laboratory for Radiology and Ultrasonography, Department and Clinic of Animal Surgery, Faculty of Veterinary Medicine of the University of Life Sciences in Lublin, ul. Głęboka 30, 20-612 Lublin, Poland; renata.komsta@up.lublin.pl; 3 Department and Clinic of Animal Surgery, Faculty of Veterinary Medicine of the University of Life Sciences in Lublin, ul. Głęboka 30, 20-612 Lublin, Poland; matszadkowski@gmail.com

**Keywords:** *Encephalitozoon cuniculi*, *Cavia porcellus*, PCR, guinea pig

## Abstract

**Simple Summary:**

Encephalitozoonosis is a disease caused by *Encephalitozoon cuniculi*. It is diagnosed primarily in rabbits, and is less frequently so in other animal species. *E. cuniculi* is classified as Microsporidia—fungi frequently found in the environment, that are resistant to numerous external factors. This pathogen is diagnosed primarily in rabbits, and is less frequently so in other animal species. The objective of the study was to analyze the prevalence of *E. cuniculi* infections in guinea pigs with different clinical disorders. The infected animals most frequently exhibited nervous and urinary system symptoms, as well as issues with vision organs, while several animals were recorded as having problems with the respiratory system and thyroid gland dysfunction. The study shows that encephalitozoonosis constitutes a significant problem in rodents kept as domestic animals, which in turn may be a source of infection for humans.

**Abstract:**

Encephalitozoonosis is a disease caused by *E. cuniculi*. It is diagnosed primarily in rabbits but is less frequently so in other animal species. *E. cuniculi* is classified among Microsporidia—fungi frequently found in the environment, that are resistant to numerous external factors. Apart from rabbits, rodents form the next group of animals most exposed to infection with these pathogens. The objective of the study was to analyze the prevalence of *E. cuniculi* infection in guinea pigs with different clinical disorders. The study included 67 animals with *E. cuniculi* infection confirmed via real-time PCR. The infected animals most frequently exhibited nervous and urinary system symptoms, as well as issues with vision organs, while several animals were also recorded as having problems with the respiratory system and thyroid gland dysfunction. The study shows that encephalitozoonosis constitutes a significant problem in rodents kept as domestic animals, which in turn may be a source of infection for humans.

## 1. Introduction

*Encephalitozoon cuniculi* is an opportunistic intracellular pathogen belonging to the Microsporidia organism related to fungi, as determined via phylogenetic analysis. Recently, some studies suggested that Microsporidia should be classified as a sister group of fungi and related to Cryptomicota [1,2,3,4,5].

The hosts of Microsporidia include vertebrates and certain protozoa [6]. The disease caused by these pathogens is prevalent in the rabbit population. The seproprevalence of *E. cuniculi* in the domestic rabbit (*Oryctolagus cuniculus domesticus*) ranges widely from 7.7% [7] to 71% [8]. Among other animal species, infection with this microorganism was found in rats, mice, muskrats, cavies, gerbils, shrews, birds, horses, goats, sheep, pigs, foxes, dogs, panthers, cats, and primates, including humans [8,9,10,11,12,13].

Rodents may also be a reservoir of *E. cuniculi* for foxes, minks, and cats. Research involving wild rodents in Poland, Czechia, and Slovakia has showed a 15% prevalence in these animals [14,15,16]. *E. cuniculi* antibodies were found in 0% to 26% of the population of cats, and in 30% of the population of dogs [17,18,19,20,21].

The course of encephalitozoonosis involves pathogens affecting the nervous system, eyes, and kidneys. In rabbits, guinea pigs, and dogs, the involvement of the nervous system dominates. In cats and rabbits, the development of ocular uveitis was recorded following infection with the discussed microorganisms.

The objective of the study was to analyze the prevalence of *E. cuniculi* infection in domestic guinea pigs (*Cavia porcellus)* in Poland with different clinical disorders.

## 2. Materials and Methods

### 2.1. Animals Used in the Study

The study included 67 guinea pigs (*Cavia porcellus*) with different clinical disorders (Table 1) having encephalitozoonosis confirmed via real-time polymerase chain reaction (real-time PCR). Guinea pigs (aged 6 months to 6 years; 29 males and 38 females) were kept in groups of 2 to 4 individuals. They came from pet stores all over Poland. The study was conducted in accordance with the EU Convention for the Protection of Animals used for Scientific Purposes (revised directive 86/609/EEC—Directive of the European Parliament and of the council on the protection of animals used for scientific purposes). The owners of the animals agreed to the use of the medical records of the guinea pigs for scientific purposes.

### 2.2. Blood Testing

Blood for hematological and molecular examinations was collected from the cephalic vein using tubes with EDTA. Hematological testing was carried out on the Exigo (Boule) analyzer. The assessed parameters were white blood cells (WBC), red blood cells (RBC), hemoglobin (HGB), hematocrit (HCT), and platelet count (PLT).

The blood for biochemical tests was collected using clotting accelerator tubes. The assessed parameters were alanine aminotransferase (ALT), aspartate aminotransferase (AST), urea, creatinine (CREA), and freeT4 (fT4), and the assessment was carried out with a BS 120 Mindray analyzer.

### 2.3. Molecular Analysis

Isolation of DNA from whole blood was performed using a Genomic Mini kit (A&A Biotechnology, Gdańsk, Poland). Isolated DNA was amplified using a real-time PCR reaction. A real-time PCR was carried out using Corbett equipment. In the reaction, the following primers were used: msp3 (5′GGAATTCACACCGCCCGTCACTAT3′), msp4a (5′CCAAGCTTATGCTTAAGTCCAAGGGGT3′), and msp4b (5′CCAAGCTTATGCTTAAGTCCAGGGAG3′). To demonstrate amplification specificity, the melting temperature of the PCR products (melting curve) was determined by gradually increasing the temperature of the reaction mixture from 70 °C to 95 °C while continuously measuring fluorescence [22].

### 2.4. Imaging Testing

A radiographic examination was carried out using a digital radiography system (Leonardo DR System). Radiographic imaging of the thorax, spine, and tympanic bullae was carried out in accordance with the standardized protocols. Radiographs were analyzed using a DICOM PACS DXR X-Ray (CareRay Version: 6.0.61-186) Acquisition Software workstation.

The abdominal ultrasound was carried out using a mikrokonvex 3–9 MHz probe and a linear 10–12 MHz probe (EsaoteMyLab Twice). During the examination, the animals were laid in dorsal recumbency.

All animals were sedated for the tomography examination with inhalation anesthesia (isoflurane), and laid in sternal recumbency. Tomography was performed using a Philips MX-16 slice unit CT scanner at a 120 kV tube voltage and 120 mA, with a slice thickness of 1 mm. The postcontrast examination was carried out following the intravenous administration of the contrast agent at a dose of 600 mgI/kg iohexol (Omnipaque 300 mgI/mL; GE Healthcare, Oslo, Norway)**.** The raw data were reconstructed in soft tissue (window level, 40 HU; window width 350 HU), brain (window level, 40 HU; window width, 120 HU), and bone (window level, 500 HU; window width, 1600 HU) algorithms. Tomographic images were reviewed and analyzed on a DICOM PACS Acquisition Software workstation (Philips IntelliSpace Portal, Philips Medical Systems Nederland B.V., Bests, The Netherlands).

### 2.5. Ophthalmological Examination

All animals underwent a detailed ophthalmic examination including slit lamp biomicroscopy (Shin Nippon; Ohira Co., Ltd., Niigata, Japan) and rebound tonometery (Tonovet; iCare, Finland). Chromatic pupillary light reflexes were assessed using BPI-50 Precision Illuminator (RetinoGraphics Inc., Norwalk, CT, USA). Following pupillary dilation with 1% tropicamide (Tropicamidum 1%; WZF Polfa S.A., Warsaw, Poland), ophthalmoscopy was conducted using a direct ophthalmoscope (Welch Allyn, New York, NY, USA) and a PanOptic ophthalmoscope (PanOptic; Welch Allyn, New York, NY, USA).

## 3. Results

A real-time PCR examination confirmed that the blood of all 67 guinea pigs had the presence of *E. cuniculi* genetic material. The Ct value was in the range of 32 to 35, and the melting point of the obtained PCR products was 77.5 °C to 78.5 °C.

The most common disorders observed in the animals were those of the urinary system, the nervous system, the visual organ, and the thyroid gland. In singular cases, symptoms were observed in the respiratory, digestive, and reproductive systems, as well as the ear.

Hematological examinations of 47 animals showed increased WBC levels. Among the animals with urinary symptoms, 17 were found to have an increased urea concentration (UREA) and 13 were found to have an elevated creatinine concentration (CREA).

### 3.1. Nervous System

Nervous system disorders were observed in 13 cases (19%); the signs were torticollis (5 animals), nystagmus (3 animals), severe apathy (2 animals), further convulsions (2 animals), and ataxia (1 animal).

### 3.2. Urinary System

Urinary system disorders were observed in 23 animals (34%) infected with *E. cuniculi*. A total of nine were found to have chronic renal failure (ultrasound examinations of the animals revealed cysts of different sizes in the kidneys and a blurring of the corticomedullary structure with different levels of development). A total of six animals exhibited cystitis characterized by pollakuria, painful urination, and hematuria. In this group of animals, the ultrasound examination showed a thickening of the urinary bladder wall and highly concentrated urine. In eight other animals, excessive accumulation of sediment in the urinary bladder was observed (four animals) which was accompanied by inflammation, while the remaining animals were diagnosed with urolithiasis.

### 3.3. Eye

Disorders in the vision organ were observed in 10 animals (15%). In four animals, corneal opacity was observed (it could not be caused by an injury or the presence of a post-injury wound), a cataract was recorded in two animals, microphthalmia was recorded in two animals, and heterotropic ossification was recorded in two other animals.

### 3.4. Thyroid Dysfunction

Among the examined animals, eight (12%) had problems with thyroid function. During the examination of the T4 blood level, a result below 2 µg/dL suggesting hypothyroidism was found in five animals, whereas a result over 4 µg/dL, indicating hyperthyroidism, was found in three animals.

### 3.5. Other

A total of 13 animals (19%) were also found to exhibit symptoms of respiratory, reproductive, and digestive system or ear disorders (Table 2).

## 4. Discussion

This article presents disorders that may accompany *E. cuniculi* infections in guinea pigs. Our own observations of these pathogens were detected in animals with different clinical disorders. A serologic survey of inbred and outbred guinea pigs from a variety of sources revealed a seroprevalence from 0% to 85% related to *E. cuniculi* [13,23]. Encephalitozoonosis may be suspected in every case of neurological symptoms, disorders of the excretory system, or disorders of the vision organ. In our own study, 34% of the animals infected with *E. cuniculi* exhibited urinary system symptoms, 19% exhibited nervous system symptoms, and 15% exhibited vision organ symptoms. The available literature, apart from a description of the cases of the neurological forms of encephalitozoonosis [24,25] and a description of the histopathology lesions in the brains of the animals infected by the pathogen [26], lacks any information on the infection of *E. cuniculi* in guinea pigs. Therefore, the present study should be treated as the first report of this type, presenting the results of research conducted on a relatively large number of individuals.

According to the observations by Ozkan [27], Künzel [28], and Csokai [29], the consequences of infection with these pathogens are the development of encephalitis and nephritis [29,30]. As many as 77.5% of animals with encephalitis and 12.5% of rabbits with interstitial nephritis were diagnosed with an *E. cuniculi* infection.

The broad infectious spectrum of *E. cuniculi* and the variable course of the disease caused by these fungi can be illustrated by the fact that cases of encephalitozoonosis were recorded in juvenile seals [30], foxes [31], minks [32], cats [33], dogs [34], squirrel monkeys [35], and bearded dragons [36]. In all these cases, the infection was accompanied by nervous system symptoms (seals, foxes, minks, cats, dogs, and squirrel monkeys) or excretory system (bearded dragons), although the presence of pathogens was also recorded in the pulmonary alveolar septa and splenic red pulp. Additionally, the observations of Juan-Sallés et al., (2006) indicate that encephalitozoonosis may have a disseminated nature [37]. These authors diagnosed disseminated encephalitozoonosis in two siblings of juvenile, cottontop tamarins (*Saguinus oedipus*) and three siblings of neonatal, emperor tamarins (S. imperator) via histologic examination, histochemical analysis, electron microscopy, and polymerase chain reaction (PCR) analysis with nucleotide sequencing. All tamarins were captive, born in zoos in North America, and died with no premonitory signs of disease. The main pathologic findings were myocarditis (4/5), hepatitis (3/5), interstitial pneumonia (3/5), skeletal myositis (3/5), meningoencephalitis (2/5), adrenalitis (2/5), tubulointerstitial nephritis (1/5), myelitis (1/5), sympathetic ganglioneuritis (1/5), and retinitis (1/5). 

The fact that the infection can assume a disseminated form may explain that in thirteen guinea pigs covered by our own observations, the development of symptoms atypical for encephalitozoonsis were recorded, such as digestive, respiratory, and reproductive system disorders, as well as ear diseases.

The available literature lacks information on the association between *E. cuniculi* infections and thyroid disturbances. These symptoms were observed in as many as 8 out of the 67 examined animals, which suggests this is not an accidental correlation. Perhaps the impaired function of thyroid results from disseminated encephalitozoonosis. On the other hand, it cannot be excluded that *E. cuniculi* infections were not the causes for the development of hypothyroidism/hyperthyroidism, but these states had developed in the animals earlier on and predisposed them to infections with microsporidia, which is similar to what was described for diabetes in mice [38]. However, the animals may constitute an *E. cuniculi* reservoir for humans.

A large number of species belonging to the phylum Microsporidia are known to be capable of infecting humans. The highest pathogenic potential is associated with three species of the genus Encephalitozoon: *E. intestinalis*, *E. cuniculi*, and *E. hellem* [39,40]. The National Institute of Allergy and Infectious Diseases classified these Microsporidia as B priority pathogens. The classification means that these organisms spread moderately easily in the environment and feature an average pathogenicity index. The Environmental Protection Agency qualified the organisms as hazardous water pollutants [41].

The group of people at the highest risk of infection include individuals with lowered immunity levels, after organ transplants [41,42,43,44,45,46], and the owners of such animals as rabbits, dogs, cats, or rodents, which may be hosts of Microsporida, excreting microbial spores through the feces and urine.

## 5. Conclusions

The diseases caused by Microsporidia remain a poorly understood topic. The microorganisms are commonly found in the environment, increasing the chances of exposure to the pathogen. Encephalitozoonosis is a disease which, despite the relatively low number of records (of which the majority concerns rabbits), poses a serious threat towards other animals, including humans. Considering that its symptoms can be variable, they are often not linked with *E. cuniculi* infections as their primary cause. Knowledge on the possible clinical course of encephalitozoonosis and identification of animals that may constitute its reservoir are significant for its efficient diagnostics, prevention, and therapy, as well as public health protection.

## Figures and Tables

**Table 1:** Number of animals with selected symptoms.

Systems	Number of Patients
Nervous system	13 (19%)
Urinary system	23 (34%)
Eye	10 (15%)
Thyroid dysfunction	8 (12%)
Other	13 (19%)
Total	67 (100%)

**Table 2:** Symptoms observed in other animals.

System Type	Number of Animals	Symptoms Observed
Respiratory system	5	Inflammation of upper respiratory tract, bronchitis, discharge from the nostrils (serous/seropurulent/purulent), and sneezing.
Digestive tract	5	Pilobezoar, obstruction, enteritis, and bacterial flora disorders.
Reproductive system	2	Ovarian cysts, pyometra, and endometriosis.
Otitis	1	Torticolis and discharge from auditory canal.

## Data Availability

Not applicable.

## References

[1] Weiss L., Vossbrinck C. (1998). Microsporidiosis: Molecular and diagnostic aspects. Adv. Parasitol..

[2] Lee S.C., Corradi N., Byrnes E.J., Torres-Martinez S., Dietrich F.S., Keeling P.J., Heitman J. (2008). Microsporidia evolved from ancestral sexual fungi. Curr. Biol..

[3] Capella-Gutiérrez S., Marcet-Houben M., Gabaldón T. (2012). Phylogenomics supports microsporidia as the earliest diverging clade of sequenced fungi. BMC Biol..

[4] Corsaro D., Walochnik J., Venditti D., Steinmann J., Müller K.D., Michel R. (2014). Microsporidia-like parasites of amoebae belong to the early fungal lineage Rozellomycota. Parasitol. Res..

[5] Keeling P.J., Burki F., Wilcox H.M., Allam B., Allen E.E., Amaral-Zettler L.A., Armbrust E.V., Archibald J.M., Bharti A.K., Bell C.J. (2014). The Marine Microbial Eukaryote Transcriptome Sequencing Project (MMETSP): Illuminating the functional diversity of eukaryotic life in the oceans through transcriptome sequencing. PLoS Biol..

[6] Weiss L.M., Becnel J.J. (2014). Microsporidia: Pathogens of Opportunity.

[7] Ozkan O., Ozkan A.T., Zafer K. (2011). Encephalitozoonosis in New Zealand rabbits and potential transmission risk. Vet. Parasitol..

[8] Valencakova A., Balent P., Petrovova E., Novotny F., Luptakova L. (2008). Encephalitozoonosis in household pet Nederland Dwarf rabbits (*Oryctolagus cuniculus*). Vet. Parasitol..

[9] Dieder E.S. (2005). Microsporidiosis: An emerging and opportunistic infection in humans and animals. Acta Trop..

[10] Ditrich O., Chrdle A., Sak B., Chmelík V., Kubále J., Dyková I., Kvác M. (2011). Causative Agent of Brain Abscess in an Immunocompetent Patient. J. Clin. Microbiol..

[11] Levcutova M., Hipikova V., Faitelzon S., Benath G., Paulik S., Levkut M. (2004). Prevalence of antibodies to Encephalitozoon cuniculi in horses in Israel. Ann. Agric. Environ. Med..

[12] Snowden K., Logan K. (1999). Molecular identification of Encephalitozoonhellemin an ostrich. Avian Dis..

[13] Wasson K., Peper R.L. (2000). Mammalian Microsporidiosis. Vet. Pathol..

[14] Muller-Doblies U.U., Herzog K., Tanner I., Mathis A., Deplazes P. (2002). First isolation and characterisation of *Encephalitozoon cuniculi* from a free-ranging rat (*Rattus norvegicus*). Vet. Parasitol..

[15] Hersteinsson P., Gunnarsson E., Hjartardottir S., Skirnisson K. (1993). Prevalence of Encephalitozoon cuniculi antibodies in terrestrial mammals in Iceland, 1986 to 1989. J. Wildl. Dis..

[16] Perec-Matysiak A., Lesnianska K., Bunkowska-Gawlik K., Condlova S., Sak B., Kvac M., Rajsky D., Hildebrand J. (2019). The opportunistic pathogen Encephalitozoon cuniculi in wild living Murinae and Arvicolinae in Central Europe. Eur. J. Protistol..

[17] Sasaki M., Yamazaki A., Haraguchi A., Tatsumi M., Ishida K., Ikadai H. (2011). Serological survey of Encephalitozoon cuniculi infection in Japanese dogs. J. Parasitol..

[18] Cray C., Rivas Y. (2013). Seroprevalence of Encephalitozoon cuniculi in dogs in the United States. J. Parasitol..

[19] Duzlu O., Yildirim A., Onder Z., Ciloglu A., Yetismis G., Inci A. (2019). Prevalence and Genotyping of Microsporidian Parasites in Dogs in Turkey: Zoonotic Concerns. J. Eukaryot. Microbiol..

[20] Hsu V., Grant D.C., Zajac A.M., Witonsky S.G., Lindsay D.S. (2011). Prevalence of IgG antibodies to Encephalitozoon cuniculi and Toxoplasma gondii in cats with and without chronic kidney disease from Virginia. Vet. Parasitol..

[21] Tsukada R., Osaka Y., Takano T., Sasaki M., Inose M., Ikadai H. (2016). Serological survey of Encephalitozoon cuniculi infection in cats in Japan. J. Vet. Med. Sci..

[22] Zietek J., Adaszek Ł., Dziegiel B., Kalinowski M., Bartnicki M., Kalinowska A., Jarosz Ł., Winiarczyk S. (2014). Diagnosis of the Encephalitozoon cuniculi infections in pet rabbits with neurological symptoms. Pol. J. Vet. Sci..

[23] Chalupský J., Vávra J., Bedrník P. (1979). Encephalitozoonosis in laboratory animals—A serological survey. Folia Parasitol..

[24] Wilczyńska A., Ziętek J., Dębiak P., Smiech A., Adaszek Ł. (2020). Encephalitozoon cuniculi invasion in the domestic cavy: A clinical case. J. Exot. Pet. Med..

[25] Wan C.H., Franklin C., Riley L.K., Hook R.R., Besch-Williford C. (1996). Diagnostic exercise: Granulomatous encephalitis in guinea pigs. Lab. Anim. Sci..

[26] Illanes O.G., Tiffani-Castiglioni E., Edwards J.F., Shadduck J.A. (1993). Spontaneous encephalitozoonosis in an experimental group of guinea pigs. J. Vet. Diagn. Investig..

[27] Ozkan O., Alcigir M.E. (2018). Encephalitozoonosis infection in a traditional rabbit farm with neurological manifestations. Vet. Parasitol..

[28] Künzel F., Joachim A. (2010). Encephalitozoonosis in rabbits. Parasitol. Res..

[29] Csokai J., Gruber A., Künzel F., Tichy A., Joachim A. (2009). Encephalitozoonosis in pet rabbits (*Oryctolagus cuniculus*): Pathohistological findings in animals with latent infection versus clinical manifestation. Parasitol. Res..

[30] Seguel M., Howerth E.W., Ritter J., Paredes E., Colegrove K., Gottdenker N. (2015). Encephalitozoonosis in 2 South American Fur Seal (*Arctocephalus australis*) Pups. Vet. Pathol..

[31] Bjerkås I., Nesland J.M. (1987). Brain and spinal cord lesions in encephalitozoonosis in the blue fox. Acta Vet. Scand..

[32] Bjerkås I. (1990). Brain and spinal cord lesions in encephalitozoonosis in mink. Acta Vet. Scand..

[33] Rebel-Bauder B., Leschnik M., Maderner A., Url A. (2011). Generalized encephalitozoonosis in a young kitten with cerebellar hypoplasia. J. Comp. Pathol..

[34] Postma G.C., Pardini L., Carnevale S., Gregnoli E., Quiroga M.A., Venturini M.C., Minatel L. (2018). Fatal canine encephalitozoonosis in Latin America, first report. Postma Vet. Parasitol. Reg. Stud. Rep..

[35] Zeman D.H., Baskin G.B. (1985). Encephalitozoonosis in squirrel monkeys (*Saimiri sciureus*). Vet. Pathol..

[36] Richter B., Csokai J., Graner I., Eisenberg T., Pantchev N., Eskens H.U., Nedorost N. (2013). Encephalitozoonosis in two inland bearded dragons (*Pogona vitticeps*). J. Comp. Pathol..

[37] Juan-Sallés C., Garner M.M., Didier E.S., Serrato S., Acevedo L.D., Ramos-Vara J.A., Nordhausen R.W., Bowers L.C., Parás A. (2006). Disseminated encephalitozoonosis in captive, juvenile, cotton-top (*Saguinus oedipus*) and neonatal emperor (*Saguinus imperator*) tamarins in North America. Vet. Pathol..

[38] Francisco Neto A., Dell’Armelina Rocha P.R., Perez E.C., Xavier J.G., Peres G.B., Spadacci-Morena D.D., Alvares-Saraiva A.M., Lallo M.A. (2017). Diabetes mellitus increases the susceptibility to encephalitozoonosis in mice. PLoS ONE.

[39] Cali A., Weiss L.M., Takvorian P.M. (2005). A review of the development of two types of human skeletal muscle infections from microsporidia associated with pathology in invertebrates and cold-blooded vertebrates. Folia Parasitol..

[40] Didier E.S., Weiss L.M. (2006). Microsporidiosis: Current status. Curr. Opin. Infect. Dis..

[41] Carlson J.R., Li L., Helton C.L., Munn R.J., Wasson K., Perez R.V., Gallay B.J., Finkbeiner W.E. (2004). Disseminated microsporidiosis in a pancreas/kidney transplant recipient. Arch. Pathol. Lab. Med..

[42] Rabodonirina M., Cotte L., Radenne S., Besada E., Trepo C. (2003). Microsporidiosis and transplantation: A retrospective study of 23 cases. J. Eukaryot. Microbiol..

[43] Teachey D.T., Russo P., Orenstein J.M., Didier E.S., Bowers C., Bunin N. (2004). Pulmonary infection with microsporidia after allogeneic bone marrow transplantation. Bone Marrow Transplant..

[44] Wesołowska M., Szetela B., Kicia M., Kopacz Ż., Sak B., Rymer W., Kváč M., Sałamatin R. (2019). Dual infection of urinary tract with Enterocytozoon bieneusi and Encephalitozoon cuniculi in HIV/AIDS patients. Ann. Parasitol..

[45] Sridhar M.S., Sharma S. (2003). Microsporidial keratoconjunctivitis in a HIV-seronegative patient treated with debridement oral itraconazole. Am. J. Ophthalmol..

[46] Theng J., Chan C., Ling M.L., Tan D. (2001). Microsporidial keratoconjunctivitis in a healthy contact lens wearer without human immunodeficiencyvirus infection. Ophthalmology.

